# Small-conductance calcium-activated potassium (SK) channels in the amygdala mediate pain-inhibiting effects of clinically available riluzole in a rat model of arthritis pain

**DOI:** 10.1186/s12990-015-0055-9

**Published:** 2015-08-28

**Authors:** Jeremy M. Thompson, Guangchen Ji, Volker Neugebauer

**Affiliations:** Department of Pharmacology and Neuroscience, School of Medicine, Texas Tech University Health Sciences Center, 3601 4th St, Lubbock, TX 79430-6592 USA; Center of Excellence for Translational Neuroscience and Therapeutics, Texas Tech University Health Sciences Center, Lubbock, TX USA

**Keywords:** Amygdala, Pain, Behavior, Vocalizations, Riluzole, Potassium channels, SK channels, BK channels, Microdialysis

## Abstract

**Background:**

Arthritis pain is an important healthcare issue with significant emotional and affective consequences. Here we focus on potentially beneficial effects of activating small-conductance calcium-activated potassium (SK) channels in the amygdala, a brain center of emotions that plays an important role in central pain modulation and processing. SK channels have been reported to regulate neuronal activity in the central amygdala (CeA, output nucleus). We tested the effects of riluzole, a clinically available drug for the treatment of amyotrophic lateral sclerosis, for the following reasons. Actions of riluzole include activation of SK channels. Evidence in the literature suggests that riluzole may have antinociceptive effects through an action in the brain but not the spinal cord. Mechanism and site of action of riluzole remain to be determined. Here we tested the hypothesis that riluzole inhibits pain behaviors by acting on SK channels in the CeA in an arthritis pain model.

**Results:**

Systemic (intraperitoneal) application of riluzole (8 mg/kg) inhibited audible (nocifensive response) and ultrasonic (averse affective response) vocalizations of adult rats with arthritis (5 h postinduction of a kaolin-carrageenan monoarthritis in the knee) but did not affect spinal withdrawal thresholds, which is consistent with a supraspinal action. Stereotaxic administration of riluzole into the CeA by microdialysis (1 mM, concentration in the microdialysis fiber, 15 min) also inhibited vocalizations, confirming the CeA as a site of action of riluzole. Stereotaxic administration of a selective SK channel blocker (apamin, 1 µM, concentration in the microdialysis fiber, 15 min) into the CeA had no effect by itself but inhibited the effect of systemic riluzole on vocalizations. Off-site administration of apamin into the basolateral amygdala (BLA) as a placement control or stereotaxic application of a selective blocker of large-conductance calcium-activated potassium (BK) channels (charybdotoxin, 1 µM, concentration in the microdialysis fiber, 15 min) into the CeA did not affect the inhibitory effects of systemically applied riluzole.

**Conclusions:**

The results suggest that riluzole can inhibit supraspinally organized pain behaviors in an arthritis model by activating SK, but not BK, channels in the amygdala (CeA but not BLA).

## Background

Pain is a multidimensional experience and includes significant emotional-affective and cognitive components in addition to its sensory aspects, all of which can have a significant impact on patient quality of life [1–3]. The amygdala, a brain area for emotions, has been shown to play a key role in emotional-affective aspects of pain, emotion-driven cognitive deficits and pain modulation in animal pain models and in human neuroimaging studies [3–6]. The amygdala neurocircuitry centered on pain processing includes the lateral-basolateral (LA-BLA) and central (CeA) nuclei [3, 4]. The LA-BLA complex receives polymodal sensory inputs from several brain regions and attaches affective information, which is then relayed to the CeA. The CeA receives purely nociceptive information through the spino-parabrachio-amygdaloid tract and serves major output functions to generate emotional responses and modulate pain-related behavior. Activity of CeA neurons correlates positively with emotional responses and anxiety-like behaviors in different pain models [3, 4]. Therefore, normalizing CeA activity is a desirable strategy to inhibit pain.

Here we focus on potentially beneficial effects of activating small-conductance calcium-activated potassium (SK) channels in the amygdala. SK channels are widely expressed throughout the nervous system, including in the amygdala; they mediate the medium-type afterhyperpolarization (mAHP) and can regulate action potential firing, excitability, and synaptic transmission [7–9]. SK channels have been implicated in peripheral [10] and spinal [11, 12] antinociceptive processes, but their role in brain mechanisms of pain remain to be determined. We tested the effects of riluzole, a clinically available drug for the treatment of amyotrophic lateral sclerosis, because actions of riluzole include activation of SK channels [13] and some evidence in the literature suggests that riluzole may have antinociceptive effects through a supraspinal rather than spinal site of action. Systemically applied riluzole had antinociceptive effects in the formalin test [14–16], in the carrageenan model of hindpaw inflammation [17], and in neuropathic pain models of chronic constriction injury [18], spinal root avulsion injury [19], cervical spondolytic myelopathy [20] and spinal cord compression [21]. In the latter study, intracerebroventricular but not intrathecal injection of riluzole was effective. Riluzole injected into the periaqueductal grey prevented nociceptive behaviors induced by capsaicin injection into the periaqueductal grey [22]. Riluzole also had anxiolytic effects in the conditioned emotional response model of anxiety [16]. A clinical study found that riluzole produced pain relief in patients with irritable bowel syndrome [23].

Here we test the hypothesis that riluzole inhibits pain behaviors in an arthritis pain model, and that SK channels in the amygdala (CeA) contribute to this effect. It should be noted that there are a number of other targets of riluzole, including inhibition of voltage-gated sodium channels, inhibition of glutamatergic transmission, and increase of glutamate uptake [13, 24–27]. Inhibition of glutamatergic signaling has been suggested as a potential mechanism of riluzole’s antinociceptive effects, but riluzole has not been directly linked to proposed interactions with NMDA receptors [26] or glutamate transporters [25]. Furthermore, behavioral effects of riluzole can be similar to those of competitive NMDA receptor antagonists, but NMDA receptor activation did not reverse riluzole effects [28]. Therefore, effects of riluzole on glutamatergic transmission could be indirect and possibly linked to its action on SK channels. In fact, SK channels have been shown to interact functionally with NMDA receptors and shunt excitatory transmission in the spinal cord, amygdala, and prefrontal cortex [12, 29, 30]. The contribution of SK channels to any pain inhibiting effects of riluzole remains to be determined.

Key novelties of this study are the site of action of riluzole in the brain, involvement of SK channels in the effects of riluzole, and pain inhibiting effects of SK channel activation in the brain (amygdala).

## Results

### Systemically applied riluzole inhibits vocalizations

Vocalizations in the audible (20 Hz–16 kHz) and ultrasonic (25 ± 4 kHz) ranges, corresponding to supraspinally organized nocifensive and averse affective responses, respectively, were evoked by brief (15 s) noxious (1000–1500 g/30 mm^2^) mechanical stimulation of the knee joint with a calibrated forceps as described previously [31–33]. Duration of vocalizations was measured for a period of 1 min, starting with the onset of the stimulus, in normal naïve rats and in another group of rats before and 5 h postinduction of a kaolin/carrageenan-induced knee joint arthritis (see “Methods”).

**Fig. 1:**
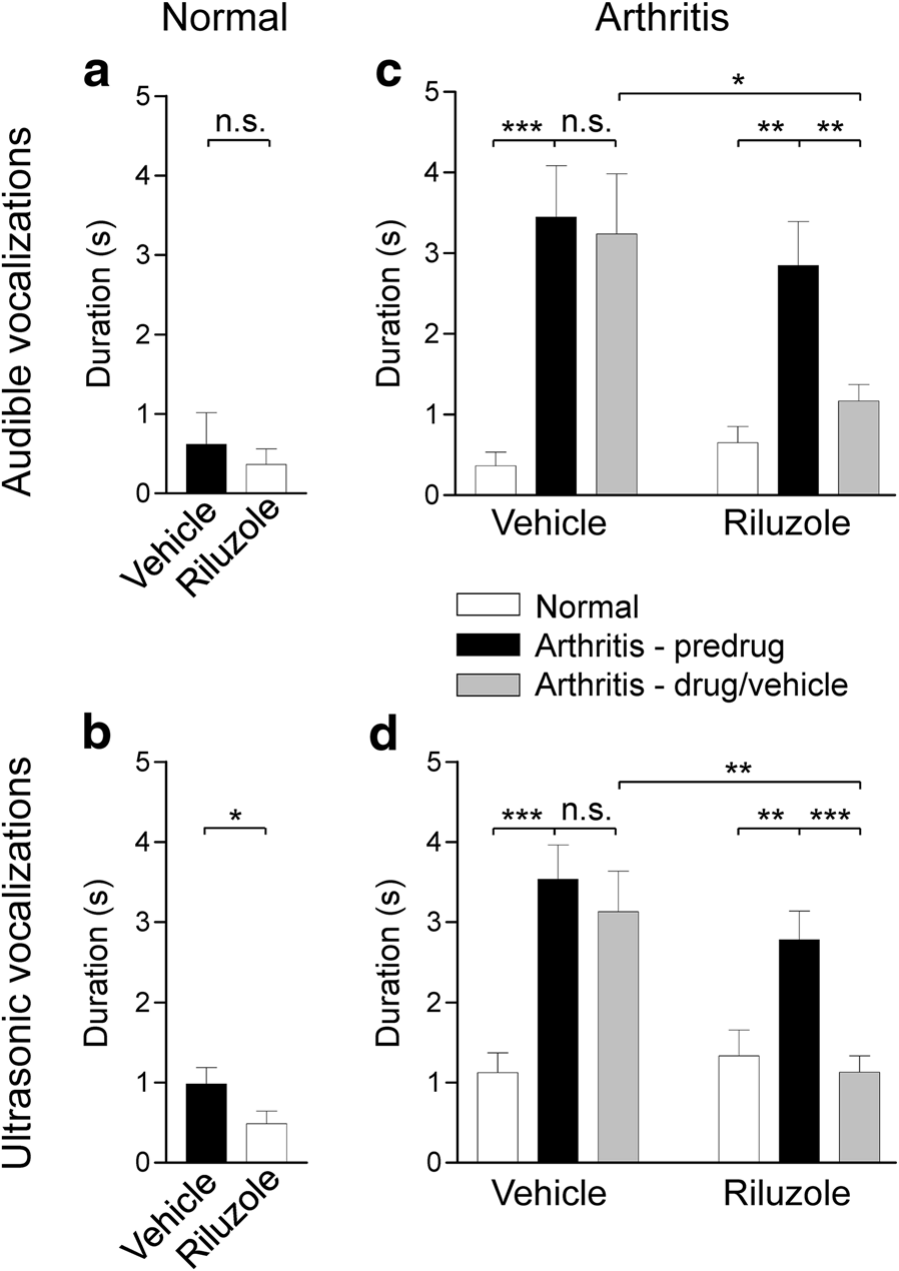
Inhibitory effects of systemically applied riluzole on vocalizations. **a**, **b** Riluzole (8 mg/kg, i.p.) had no effect on audible vocalizations (**a**) but inhibited ultrasonic vocalizations (**b**) to noxious stimulation of the knee joint compared to vehicle in normal naïve rats (n = 6). *n.s.* non-significant; *P < 0.05; paired t test. **c**, **d** Induction of arthritis resulted in a significant increase of audible (**c**) and ultrasonic (**d**) vocalizations evoked by noxious stimuli. Riluzole (8 mg/kg, i.p.; n = 19 rats) inhibited vocalizations of arthritic rats compared to predrug and vehicle (HBC, 30 %, i.p.; n = 16 rats). *n.s.* non-significant; *^,^**^,^***P < 0.05, 0.01, 0.001; repeated measures one-way ANOVA (compared to predrug) and unpaired t test (compared to vehicle) with Bonferroni posttests/correction. *Bar histograms* show mean ± SEM

In normal naïve rats, systemic injection of riluzole (8 mg/kg, i.p.; n = 6 rats) decreased the duration of ultrasonic (Fig. 1b) but not audible (Fig. 1a) vocalizations evoked by noxious stimulation of the knee joint compared to vehicle (2-hydroxypropyl-β-cyclodextrin, HBC, 30 %, i.p.) tested in the same rats (P < 0.05, paired *t* test). Induction of arthritis resulted in a significant increase in the duration of audible (Fig. 1c) and ultrasonic (Fig. 1d) vocalizations compared to normal (P < 0.001, repeated measures one-way ANOVA with Bonferroni posttests). Systemic application of riluzole (8 mg/kg, i.p.; n = 19 rats) decreased the vocalizations of arthritic rats compared to predrug and vehicle (HBC, 30 %, i.p.; n = 16 rats) significantly (P < 0.05–0.001, repeated measures one-way ANOVA and unpaired t test with Bonferroni correction/posttests).

The dose of 8 mg/kg was chosen because a previous study showed that this was the lowest dose that produced antinociceptive effects in a spinal cord injury model [21]. Vocalizations were measured 60 min postinjection of riluzole because preliminary data indicated a maximum effect of riluzole at this time point when tested every 30 min for 3 h. In a subset of rats (n = 6) that were tested 1 h and 2.5 h after systemic application of riluzole (8 mg/kg, i.p.), we found that the inhibitory effects of riluzole on audible and ultrasonic vocalizations had disappeared 2.5 h post-injection compared to vehicle control values.

### Riluzole has no effect on hindlimb withdrawal responses

**Fig. 2:**
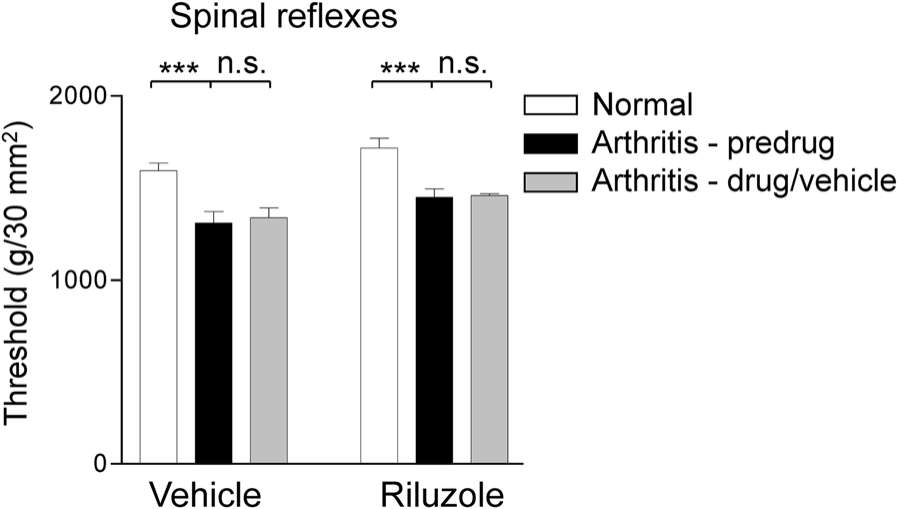
Lack of effect of systemically applied riluzole on spinal withdrawal thresholds. Induction of arthritis significantly reduced hindlimb withdrawal thresholds measured by mechanical compression of the knee joint. Systemic application of vehicle (HBC, 30 %, i.p.; n = 11 rats) or riluzole (8 mg/kg, i.p.; n = 7 rats) had no effect compared to predrug values. *Bar histograms* show mean ± SEM. *n.s.* non-significant; ***P < 0.001; repeated measures one-way ANOVA with Bonferroni posttests

Hindlimb withdrawal thresholds were measured by compressing the knee joint with gradually increasing force using a calibrated forceps whose output was displayed on an LCD screen until a withdrawal response was observed as described in our previous studies [31, 33] (see “Methods”). Reflex thresholds were significantly decreased 5 h postinduction of arthritis (P < 0.001, repeated measures one-way ANOVA with Bonferroni posttests; Fig. 2). Systemic application of riluzole (8 mg/kg, i.p.; n = 7 rats) or vehicle (HBC, 30 %, i.p.; n = 11) had no effect compared to predrug control values (P > 0.05; repeated measures one-way ANOVA with Bonferroni posttests; Fig. 2).

### Riluzole inhibits vocalizations through an action on SK channels in the CeA but not BLA

**Fig. 3:**
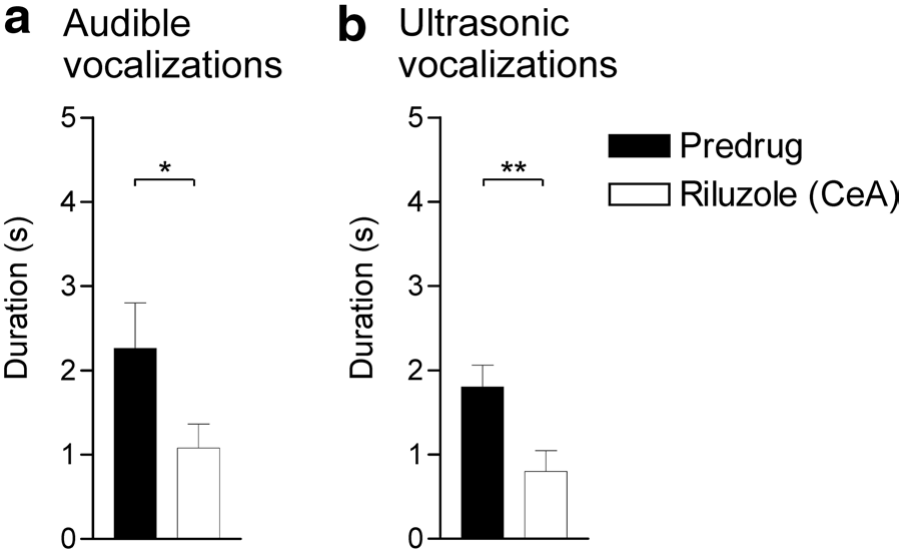
Inhibitory effects of riluzole administered into the amygdala (CeA) in arthritic rats. Stereotaxic administration of riluzole (1 mM, concentration in the microdialysis probe, 15 min) into the CeA of arthritic rats (n = 8 rats; 5 h postinduction) significantly inhibited audible (**a**) and ultrasonic (**b**) vocalizations evoked by noxious stimulation of the knee joint compared to predrug values. *Bar histograms* show mean ± SEM. *^,^**P < 0.05, 0.01; paired t test

**Fig. 4:**
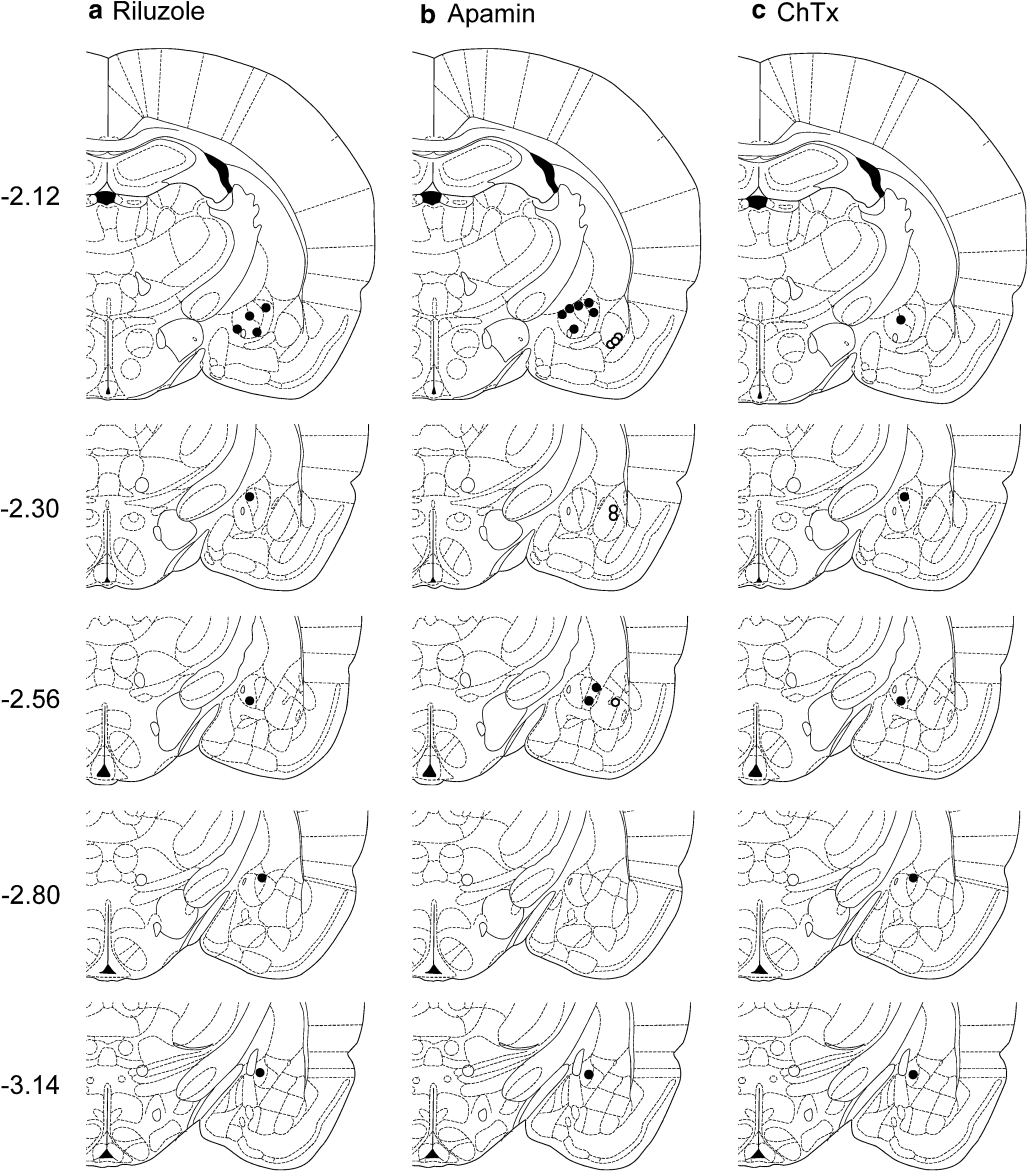
Location of microdialysis probes for stereotaxic drug application. *Symbols* show the positions of the tips of microdialysis probes for drug application into amygdala regions. CeA, *filled circles*; BLA, *open circles*. **a** Positions of microdialysis probes for stereotaxic application of riluzole into the CeA. **b** Positions of microdialysis probes for stereotaxic application of apamin into CeA or BLA; *gray symbols* indicate sites of apamin injection that increased vocalizations. **c** Positions of microdialysis probes for stereotaxic application of charybdotoxin (ChTx) into CeA or BLA. *Diagrams* show coronal brain slices. *Numbers* indicate distance from the bregma

To determine site of action in the brain, we injected riluzole (1 mM, concentration in the microdialysis fiber, 15 min) stereotaxically into the central nucleus of the amygdala (CeA) by microdialysis and measured its effect on vocalizations of arthritic rats (5 h postinduction). Microdialysis has been used routinely in our previous studies for drug application into different brain areas (for recent publications see [32–34]). Riluzole administered into the CeA significantly decreased the duration of audible (Fig. 3a) and ultrasonic (Fig. 3b) vocalizations evoked by noxious stimulation of the knee compared to predrug values (n = 8 rats; P < 0.05–0.01; paired t test). Position of microdialysis probes in the CeA was verified histologically (Fig. 4a).

**Fig. 5:**
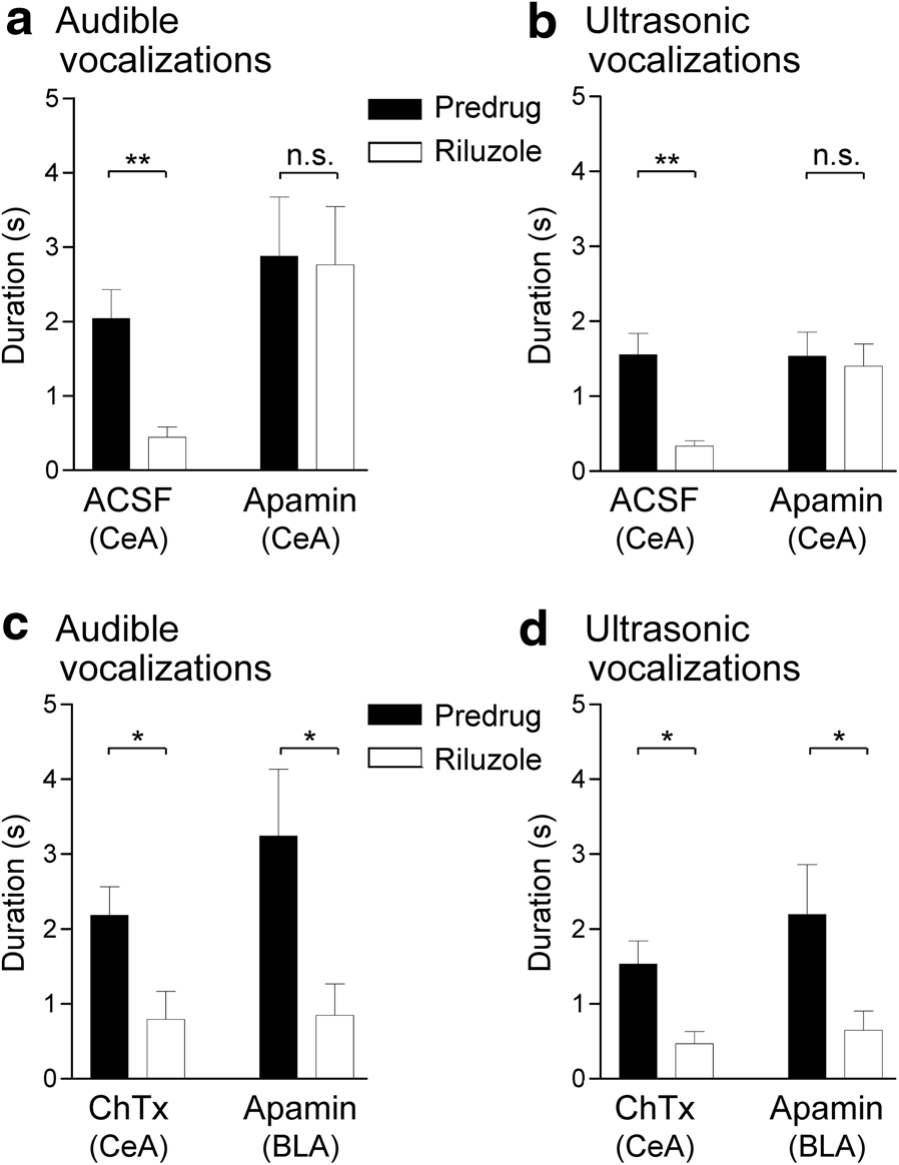
Involvement of SK, but not BK, channels in the CeA, but not BLA, in the inhibitory effects of riluzole in arthritis. **a**, **b** Systemic riluzole (8 mg/kg, i.p.) had no effect on audible (**a**) and ultrasonic (**b**) vocalizations compared to predrug values, when an SK channel blocker (apamin, 1 μM, concentration in the microdialysis probe, 15 min) was administered stereotaxically into the CeA of arthritic rats (n = 9 rats; 5 h postinduction). When ACSF was administered into the CeA, systemic riluzole inhibited vocalizations of arthritic rats significantly compared to predrug values (n = 9 rats). *n.s.* non-significant; **P < 0.01; paired t test. **c**, **d** Stereotaxic application of a BK channel blocker (charybdotoxin, ChTx, 1 μM, concentration in the microdialysis probe, 15 min) into the CeA (n = 5 rats) or stereotaxic application of apamin (1 μM, concentration in the microdialysis probe, 15 min) into the BLA (n = 6 rats) did not block the significant inhibitory effects of systemic riluzole on audible (**c**) and ultrasonic (**d**) vocalizations of arthritic rats compared to predrug values. *n.s.* non-significant; *^,^**P < 0.05; paired t test. *Bar histograms* show mean ± SEM

To assess the contribution of SK channels in the CeA to the inhibitory effects of riluzole, a selective SK channel blocker (apamin, 1 μM, concentration in the microdialysis fiber, 15 min) was stereotaxically applied into the CeA of arthritic rats (5 h postinduction), starting 45 min after systemic injection of riluzole (8 mg/kg, i.p.). Vocalizations were measured 15 min later, i.e., at 1 h post-injection of riluzole. In the presence of apamin in the CeA, systemically applied riluzole had no effect on audible (Fig. 5a) or ultrasonic (Fig. 5b) vocalizations compared to predrug control values (n = 9 rats; P > 0.05; paired t test). In contrast, stereotaxic administration of artificial cerebrospinal fluid (ACSF, 15 min) into the CeA as a control did not block the effect of systemically applied riluzole, and so riluzole was able to inhibit audible (Fig. 5a) and ultrasonic (Fig. 5b) vocalizations compared to predrug control values (n = 9 rats; P < 0.01; paired t test). Position of microdialysis probes in the CeA was verified histologically (Fig. 4b).

To determine if the involvement of SK channels was specific to the CeA, placement control experiments were performed in arthritic rats (5 h postinduction) and apamin (1 μM, concentration in the microdialysis probe, 15 min) was administered stereotaxically into the BLA, which is a major source of input to the CeA [3]. Apamin administration started 45 min after systemic application of riluzole (8 mg/kg, i.p.) and vocalizations were measured 15 min later, i.e., at 1 h post-injection of riluzole. Apamin administered into the BLA did not block the significant inhibitory effects of riluzole (n = 6 rats; P < 0.05, compared to predrug, paired t test) on audible (Fig. 5c) and ultrasonic (Fig. 5d) vocalizations. Position of microdialysis probes in the BLA was verified histologically (Fig. 4b).

### BK channels in the CeA are not involved in the effects of riluzole

To assess a contribution of large-conductance calcium-activated potassium (BK) channels to the inhibitory effects of riluzole, a selective BK channel blocker (charybdotoxin, 1 μM, concentration in the microdialysis probe, 15 min) was administered stereotaxically into the CeA of arthritic rats (n = 5 rats; 5 h postinduction), starting 45 min after systemic injection of riluzole (8 mg/kg, i.p.). Vocalizations were measured 15 min later, i.e., at 1 h post-injection of riluzole. Charybdotoxin did not affect the effects of systemically applied riluzole and so riluzole inhibited audible (Fig. 5c) and ultrasonic (Fig. 5d) vocalizations significantly compared to predrug control values (P < 0.05; paired t test). Position of microdialysis probes in the CeA was verified histologically (Fig. 4c).

### SK channel blockade in the CeA has no effect on vocalizations and hindlimb withdrawal responses

**Fig. 6:**
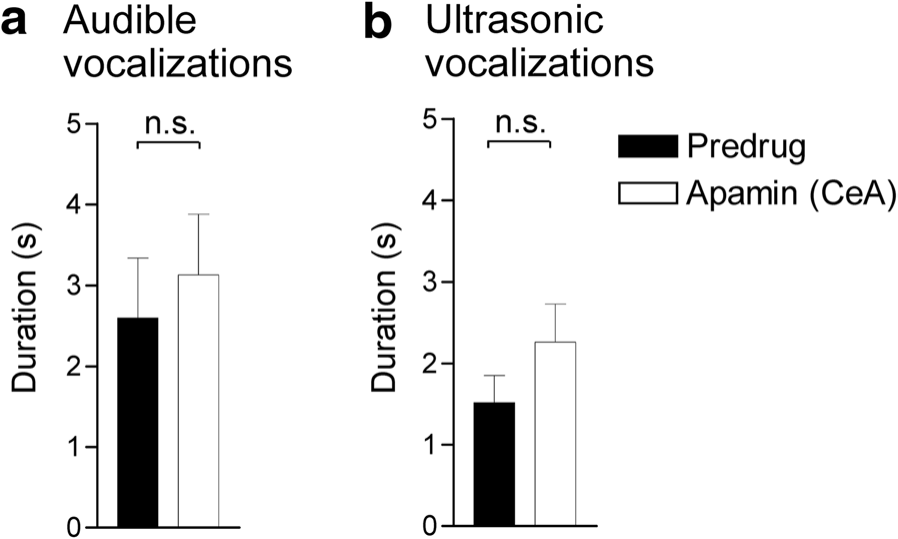
Lack of effect of SK channel blockade alone on vocalizations in arthritic rats. Stereotaxic application of a selective SK channel blocker (apamin, 1 μM, concentration in the microdialysis probe, 15 min) into the CeA alone had no significant effect on audible (**a**) and ultrasonic (**b**) vocalizations of arthritic rats (n = 7 rats; 5 h postinduction) compared to predrug values. *Bar histograms* show mean ± SEM. *n.s.* non-significant; paired t test

The data so far show an important contribution of SK, but not BK, channels in the CeA, but not BLA, to the inhibitory effects of riluzole. To assess the role of SK channels in pain-related behaviors, we measured the effect of apamin (1 μM, concentration in the microdialysis probe, 15 min) administered stereotaxically into the CeA on vocalizations of arthritic rats (n = 7). Apamin by itself had no significant effect on audible (Fig. 6a) or ultrasonic (Fig. 6b) vocalizations, or on hindlimb withdrawal thresholds (data not shown), compared to predrug control values (P > 0.05; paired t test), but there was a trend because some rats showed increased vocalizations during apamin application (audible, three rats; ultrasonic, four rats; see gray symbols in Fig. 4b). Position of microdialysis probes in the CeA was verified histologically (Fig. 4b).

## Discussion

Key findings of this study are that riluzole is antinociceptive in an arthritis pain model and that this effect involves activation of small-conductance calcium-activated potassium (SK) channels, but not large-conductance calcium-activated potassium (BK) channels, in the central nucleus of the amygdala (CeA). To the best of our knowledge, this is the first study to show that SK channel activation in the amygdala can inhibit pain-related behaviors. These findings are significant because they demonstrate preclinical efficacy of a clinically-available drug (riluzole) for the treatment of pain, provide information about mechanisms and site of action, and suggest that SK channels in the amygdala could be targeted for pain management.

Riluzole is a clinically available compound approved by the US Food and Drug Administration (FDA) for the treatment of amyotrophic lateral sclerosis (ALS). Riluzole is currently in clinical trials for a phase 2/3 study on spinal cord injury treatment (“Riluzole in Spinal Cord Injury Study”, RISCIS; clinicaltrials.gov Identifier: NCT01597518), a phase 1/2 study on post-traumatic stress disorder [“Safety Study of Riluzole to Treat Post-traumatic Stress Disorder (PTSD)”; ClinicalTrials.gov Identifier: NCT02155829], and a phase 2 study for depression (“efficacy and tolerability of riluzole in treatment resistant depression”; ClinicalTrials.gov Identifier: NCT01204918). Riluzole has been investigated in preclinical and clinical studies for beneficial effects in pain. Riluzole had antinociceptive effects in rodent models of inflammatory [14, 16, 17] and neuropathic pain [18–21] and produced pain relief in patients with irritable bowel syndrome [23] and a slight pain reduction in patients with peripheral neuropathic pain, though no overall significant effect compared to placebo [35]. A study in healthy volunteers reported a reduction of median pain during thermal injury on the distal leg at the time point when maximum plasma concentrations of riluzole were expected (90 min after oral application), but riluzole had no analgesic effects in normal skin or on mechanical and thermal cutaneous hypersensitivity hours after burn injury [36].

Some preclinical evidence pointed to a supraspinal site of action of riluzole. Intracerebroventricular, but not intrathecal, injection of riluzole was effective in neuropathic spinal cord injury pain [21], although another study found that systemically applied riluzole inhibited spinal glutamate release in the formalin pain model [15]. Antinociceptive effects of riluzole in a carrageenan-induced inflammatory pain model were accompanied by decreased concentrations of glutamate and aspartate in the thalamus [17]. Riluzole injected into the periaqueductal grey prevented capsaicin-induced nociceptive behaviors [22]. The site of antinociceptive action of riluzole is not clear yet. Here we identified the amygdala (CeA) and SK channels as a site and mechanism of action of riluzole in an arthritis pain model. Systemic administration of riluzole resulted in a significant attenuation of supraspinally organized vocalizations but not hindlimb withdrawal responses. Lack of effects on withdrawal thresholds argues against motor deficits and non-specific generalized effects such as a local anesthetic action from sodium channel blockade.

The results of the present study identify the amygdala as a site of action because stereotaxic application of riluzole into the CeA inhibited pain behaviors and intra-CeA application of an SK channel blocker (apamin) prevented the antinociceptive effects of systemic riluzole. These effects were specific for SK channels in the CeA because they were not mimicked by apamin application into another amygdala area (BLA) or by application of a BK channel blocker (charybdotoxin) into the CeA, and so these findings further argue against non-specific actions. It should be noted that functional SK channels contributing to the medium AHP have been reported in the lateral amygdala area, but they do not regulate action potential firing frequency [37]. Therefore, it is possible that apamin-induced block of SK channels in the lateral-basolateral area of the amygdala did not translate into a significant effect on amygdala output from the CeA because SK channels do not regulate BLA neuronal firing. It should be noted that the present study does not rule out contributions of other brain regions or other molecular targets to the antinociceptive effects of riluzole. SK channel blockade in the CeA by itself (without riluzole), had no significant behavioral effects in the pain model, perhaps suggesting that SK channels are not endogenously activated or are not sufficient to control amygdala-dependent behaviors. Furthermore, this finding suggests that the reversal of inhibitory effects of systemic riluzole by intra-CeA apamin was not due to an indirect effect but instead a specific blockade of riluzole actions. There was a trend for apamin to increase vocalizations in about half of the sample of rats and so there could be inter-individual variability involving SK channels. Comparison of the injection sites that increased vocalizations and those that had no effect did not show evidence for site specific actions within the CeA.

While this study was motivated in part by the clinical availability of a compound (riluzole) that acts on SK channels, inhibition of glutamate signaling could also mediate the antinociceptive effects [24–26], although this may be an indirect effect of riluzole and could be the consequence of SK channel activation such as shunting excitatory transmission [37]. SK channels have been shown to modulate NMDA receptor mediated currents, indicating that SK channel activation could be responsible for the indirect inhibition of glutamate function observed with riluzole [30, 38]. Activation of SK channels in the spinal cord had antinociceptive effects and reduced the dose of an NMDA receptor blocker needed to attenuate mechanical hypersensitivity in an inflammatory pain model [12]. Data from the present study suggest that amygdala-mediated actions of riluzole on pain-related behaviors depend on SK channels. Pain-related consequences and downstream effects of SK channel activation remain to be determined.

Our studies rely on the choice of appropriate concentrations for systemic and focal (by microdialysis) application. The dose of systemically applied riluzole (8 mg/kg) has been shown to have antinociceptive effects in previous studies [15, 20, 21]. For stereotaxic drug application by microdialysis, a concentration 100-fold greater than that needed in the tissue was selected based on published data (riluzole, [13]; apamin, [37]; charybdotoxin, [39]). Our previous studies comparing drug effects in brain slices with drug application by microdialysis in intact animals determined that microdialysis required a 100-fold higher concentration because of the concentration gradient across the dialysis membrane and diffusion in the tissue [32, 40–43]. Riluzole, being a small molecule, might be expected to have a higher transfer efficiency across the microdialysis membrane than the comparatively larger peptides apamin and charybdotoxin. However, previous studies with stereotaxic application of similar small molecules and peptides have indicated that the 100-fold concentration gradient is appropriate for transfer in both situations [32, 41, 43]. However, we cannot rule completely that reduced transfer efficiency contributed to the inability of charybdotoxin to block the inhibitory effects of systemic riluzole.

A number of other questions arise from the results of this behavioral study that warrant further research. While our data implicate SK channels in the inhibitory effects of riluzole, the role of SK channels in the amygdala in pain remains to be determined. Behavioral and electrophysiological effects of selective SK channel activators and synaptic and cellular mechanisms and site(s) of action of SK channels should be investigated. Here we focused on the effects of riluzole because it is a clinically available compound. Detailed electrophysiological analysis of its actions in the amygdala circuitry is needed. And while the present study focused on emotional responses in a relatively acute pain model (knee joint arthritis), it would be important to measure effectiveness of riluzole in more chronic models and on other parameters such as anxiety and depression.

## Conclusions

SK channel activation in the amygdala (CeA) plays an important role in the antinociceptive effects of riluzole in an arthritis pain model, suggesting that SK channels in that brain area may be useful targets for pain relief and are accessible to systemically administered clinically available compounds. To the best of our knowledge this is the first study to link SK channels in the amygdala to pain modulation.

## Methods

Adult male Sprague–Dawley rats (180–350 g) were housed in a temperature-controlled room and maintained on a 12-h day/night cycle with unrestricted access to food and water. Experimental procedures were approved by the Institutional Animal Care and Use Committee (IACUC) at Texas Tech University Health Sciences Center.

### Experimental protocol

Pain behaviors were measured before and 5 h after induction of a mono-arthritis in the left knee joint. To test the effects of systemic (intraperitoneal, i.p.) application of riluzole, pain behaviors (see “Pain-related behaviors”) were measured 1 h postinjection in normal and arthritic animals. To determine effects of drug application into the amygdala, pain behaviors were measured 15 min after starting drug application through a stereotaxically implanted microdialysis probe. To investigate site of action in the amygdala of systemically applied riluzole, potassium channel blockers (or ACSF) were administered into the amygdala 45 min after systemic application of riluzole and pain behaviors were measured 15 min later, i.e., 1 h postinjection of riluzole (i.p.).

### Arthritis pain model

Rats were briefly anesthetized with isoflurane (2–3 %) and a mono-arthritis was induced by injections of kaolin (4 %, 100 μl) and carrageenan (2 %, 100 μl) into the left knee joint cavity through the patellar ligament followed by repetitive flexion and extension of the leg for several minutes after each injection [31]. Testing began 5 h post-injections because electrophysiological and behavioral changes reach a plateau at this time point [31].

### Stereotaxic drug application into the amygdala by microdialysis

These procedures have been described in our previous studies (for recent publications see [32, 33, 44]). On Day 1, a guide cannula was inserted into the right CeA. Rats were anesthetized with isoflurane (2–3 %) and placed in a stereotaxic frame (David Kopf Instruments). After a unilateral craniotomy, a guide cannula (CMA/Microdialysis, Solna, Sweden) was stereotaxically inserted into the right CeA using the following coordinates [45]: 2.1 mm caudal to bregma, 4 mm lateral to midline, and 7 mm deep. The cannula was fixed to the skull with dental acrylic (Plastic One, Roanoke, VA, USA). Bacitracin ointment was applied to the exposed tissue to prevent infection. For offsite control injections, cannulas were implanted lateral to the CeA into the BLA (see Fig. 4b). On Day 2, a microdialysis probe (CMA/Microdialysis 11, Solna, Sweden) extending 1 mm beyond the cannula was inserted and connected to an infusion pump (Harvard Apparatus, Holliston, MA, USA) using polyethylene tubing. Drug (riluzole, apamin, or ChTx) or vehicle (ACSF) was applied for 15 min at a rate of 5 μl/min before behavioral testing to establish tissue equilibrium. Location of the tips of the microdialysis probes were verified histologically (see “Histological verification of injection sites”).

### Pain-related behaviors

#### Vocalizations

Vocalizations in the audible (20 Hz–16 kHz) and ultrasonic (25 ± 4 kHz) ranges were measured using a condenser microphone connected to a preamplifier, and bat detector connected to a filter and amplifier (UltraVox 4-channel system; Noldus Information Technology), respectively, as described previously [31, 46]. Rats were briefly anesthetized with isoflurane and positioned comfortably in a custom-designed recording chamber (US Patent 7,213,538) at a fixed distance from the sound detectors. Brief (15 s) innocuous (100–500 g/30 mm^2^) and noxious (1000–1500 g/30 mm^2^) stimuli were applied to the knee with a calibrated forceps equipped with a force transducer whose output signal was amplified and displayed in grams on an LED screen. Vocalizations were recorded for 1 min starting with the onset of the mechanical stimulus and analyzed using Ultravox 2.0 software (Noldus Information Technology).

#### Hindlimb withdrawal responses

Withdrawal thresholds were determined after the vocalization assay as described previously [31]. A calibrated forceps with force transducer was used to apply a mechanical stimulus of continuously increasing intensity to the left knee joint until a withdrawal reflex was evoked. Withdrawal threshold was defined as the force required for a reflex response.

### Histological verification of injection sites

The position of the microdialysis probe was confirmed histologically. Animals were euthanized by decapitation using a guillotine (Harvard Apparatus Decapitator) at the end of the experiment, and brains were removed rapidly and submerged in 4 % paraformaldehyde at 4 °C for 24 h. Tissues were stored in 30 % sucrose and frozen sectioned at 50 μm. Sections were mounted on gel-coated glass slides and stained with hematoxylin and eosin (H&E) before cover slipping. Sections were analyzed under the microscope and positions of the tip of the probes were identified and plotted on diagrams adapted from [45].

### Drugs

The following drugs were used: riluzole [2-amino-6-(trifluoromethoxy)benzothiazole] was purchased from Sigma-Aldrich and dissolved in 2-hydroxypropyl-β-cyclodextrin (HBC, Sigma-Aldrich; 30 %), which served as the vehicle control for systemic drug injection. Total volume was increased to 1 ml with 0.9 % isotonic saline for i.p. injections (containing <10 % HBC). For stereotaxic administration by microdialysis, riluzole was diluted in ACSF from a stock solution made with HBC (30 %). Apamin (SK channel blocker) and charybdotoxin (BK channel blocker) were purchased from Tocris Bioscience (R&D Systems, Minneapolis, MN, USA). For stereotaxic administration by microdialysis, apamin and charybdotoxin were dissolved ACSF, which served as a vehicle control.

### Statistics

Statistical significance was accepted at the level P < 0.05. All averaged values are presented as means ± SEM. GraphPad Prism 3.0 software (Graph-Pad Software, San Diego, CA, USA) was used for analysis. Repeated measures one-way ANOVA with Bonferroni posttests and paired t tests were used where appropriate.

## Abbreviations

ACSF: artificial cerebrospinal fluid
AHP: afterhyperpolarization
ALS: amyotrophic lateral sclerosis
BK channel: large-conductance calcium-activated potassium channel
BLA: basolateral nucleus of the amygdala
CeA: central nucleus of the amygdala
ChTx: charybdotoxin
HBC: 2-hydroxypropyl-β-cyclodextrin
i.p.: intraperitoneal
LA: lateral nucleus of the amygdala
SK channel: small-conductance calcium-activated potassium channel
